# Supplementary material for: A novel DNA primase-helicase pair encoded by SCCmec elements
Source: eLife. 2020 Sep 18;9:e55478. doi: 10.7554/eLife.55478 (PMC7581432; doi:10.7554/eLife.55478)
Supplement: Supplementary file 1. — Table 1. Primers used in the study. Table 2. Oligonucleotides used for in vitro assays. Table 3. Primase start sites identified in the study. [file elife-55478-supp1.docx]

**Supplementary Table 1:** Primers used in the study.

| **Primer** | **Sequence** | **Purpose** |
| --- | --- | --- |
| 1 | TATGTATCAAGCAAATATACGTG | Amplification of CCPol (for1) |
| 2 | TGTATCAAGCAAATATACGTG | Amplification of CCPol (for2) |
| 3 | GTTCATTGTCTATACCTCC | Amplification of CCPol (rev1) |
| 4 | TCGAGTTCATTGTCTATACCTCC | Amplification of CCPol (rev2) |
| 5 | GATCGGATCCATGAATCATATTTTAGA | Amplification of Cch2 (for) |
| 6 | GATCCTCGAGTCATTATTCATCGTCAAAGTCC | Amplification of Cch2 (rev) |
| 7 | ATTTTGTTTAACTTTAAGAAGGAGATATACCATGGGCAGCAGCCATCATCATCA | RF cloning of N-term 6xHis-Thrombin into Cch2 expression vector (for) |
| 8 | AATAATTTTATTAACATTTCTAAAATATGATTCATCATATGGCTGCCGCGCGG | RF cloning of N-term 6xHis-Thrombin into Cch2 expression vector (rev) |
| 9 | ATATGATTCATCATATGCGCTTGGAAGTACAGGTTTTCGCTGCCGCGCGGCACC | Introduction of TEV cleavage site into Cch2 expression vector (for) |
| 10 | GGTGCCGCGCGGCAGCGAAAACCTGTACTTCCAAGCGCATATGATGAATCATAT | Introduction of TEV cleavage site into Cch2 expression vector (rev) |
| 11 | CGAAAACCTGTACTTCCAAGCGCATATGATGTATCAAGCAAATATACGTGATT | RF cloning of Cch2 operon (for) |
| 12 | GGTGGTGGTGGTGCTCGAGTTATTCATCGTCAAAGTCCTCC | RF cloning of Cch2 operon (rev) |
| 13 | GATCCTCGAGGCTAGCTCATTATTCATCGTCAAAGTCC | Amplification of Cch2 for polycistronic cloning + 6xHis-SUMO (rev) |
| 14 | GATCGGATCCATGAATAATGATAGAGGAAAAAGTCTTC | Amplification of MP for polycistronic cloning + 6xHis-SUMO (for) |
| 15 | GATCCTCGAGGCTAGCTTACAATACCCCTCCAAATTCATC | Amplification of MP for polycistronic cloning + 6xHis-SUMO (rev) |
| 16 | GATCGGATCCATGTATCAAGCAAATATACGTGATTTAATTAC | Amplification of CCPol for polycistronic cloning + 6xHis-SUMO (for) |
| 17 | GATCCTCGAGGCTAGCTTATTCATTGTCTATACCTCCTAAAGC | Amplification of CCPol for polycistronic cloning + 6xHis-SUMO (rev) |
| 18 | GATCCCATGGATGAATCATATTTTAGAAATG | Amplification of Cch2 for polycistronic cloning - 6xHis-SUMO (for) |
| 19 | GATCGGATCCGCTAGCTCATTATTCATCGTCAAAGTCC | Amplification of Cch2 for polycistronic cloning - 6xHis-SUMO (rev) |
| 20 | GATCCCATGGATGAATAATGATAGAGGAAAAAGTCTTC | Amplification of MP for polycistronic cloning - 6xHis-SUMO (for) |
| 21 | GATCGGATCCGCTAGCTTACAATACCCCTCCAAATTCATC | Amplification of MP for polycistronic cloning - 6xHis-SUMO (rev) |
| 22 | GATCCCATGGATGTATCAAGCAAATATACGTGATTTAATTAC | Amplification of CCPol for polycistronic cloning - 6xHis-SUMO (for) |
| 23 | GATCGGATCCGCTAGCTTATTCATTGTCTATACCTCCTAAAGC | Amplification of CCPol for polycistronic cloning - 6xHis-SUMO (rev) |
| 24 | GCAACTGATGGTTCGAATGATTTAAACGCTATTGTATAAACCTTTTTAAATTGCG | CCPol mutagenesis: D169A (for) |
| 25 | CGCAATTTAAAAAGGTTTATACAATAGCGTTTAAATCATTCGAACCATCAGTTGC | CCPol mutagenesis: D169A (rev) |
| 26 | CATTACATTCAATCATAATCGCCGCGTGTATCGGCACTATAATCTTG | CCPol mutagenesis: D324A (for) |
| 27 | CAAGATTATAGTGCCGATACACGCGGCGATTATGATTGAATGTAATG | CCPol mutagenesis: D324A (rev) |
| 28 | GACTTAACTAATTTAATATAGACCGATTCCCCATTTTTTGCAGTTCCATATAAAAAAAGTCC | Cch2 mutagenesis: K252E (for) |
| 29 | GGACTTTTTTTATATGGAACTGCAAAAAATGGGGAATCGGTCTATATTAAATTAGTTAAGTC | Cch2 mutagenesis: K252E (rev) |
| 30 | GATCGGATCCTTGGGAGGACTTTGACGATGAATAATG | Amplification of putative ori (for) |
| 31 | GATCGAATTCCGTGAATAAAGTGCAATTTTACCCTTCATATC | Amplification of putative ori (rev) |
| 32 | ATATTTATAATTTCGTTT AATCATATGAGTACTACAGGCAACTGCCTGTAG | Deletion of DR from putative ori (for) |
| 33 | CTACAGGCAGTTGCCTGTAGTACTCATATGATT AAACGAAATTATAAATAT | Deletion of DR from putative ori (rev) |
| 34 | TTTTTATCACTTTTACCACTTAATCATATGAGTAATTAGCCTTAATTACCTTTATTGATACG | Deletion of IR from putative ori (for) |
| 35 | CGTATCAATAAAGGTAATTAAGGCTAATTACTCATATGATTAAGTGGTAAAAGTGATAAAAA | Deletion of IR from putative ori (rev) |
| 36 | TATACCATGGGCCTTGTTCC | Amplification of Random 1 (for) |
| 37 | ACGCCTTGATAACCCCTTTC | Amplification of Random 1 (rev) |
| 38 | CGGAGGTGGATGAAGAGAAA | Amplification of Random 2 (for) |
| 39 | TGCTGCGGACAATCTAAATG | Amplification of Random 2 (rev) |
| 40 | GAGATAACAGTACGTGATTTTAAGAAAGTGGTAAAAGTGATAAAAATGCCTTGGCACCACG | Addition of DR to Random 1 (for) |
| 41 | CGTGGTGCCAAGGCATTTTTATCACTTTTACCACTTTCTTAAAATCACGTACTGTTATCTC | Addition of DR to Random 1 (rev) |
| 42 | CATTCGATAATGAATCTACGCATTTTTATCACTTTTACCACTTTAACCTATAGTTTTCTTTTTTAAATCAATTATGCCCAAC | Addition of DR to Random 2 (for) |
| 43 | GTTGGGCATAATTGATTTAAAAAAGAAAACTATAGGTTAAAGTGGTAAAAGTGATAAAAATGCGTAGATTCATTATCGAATG | Addition of DR to Random 2 (rev) |

All sequences shown in 5’ to 3’ direction. Introduced restriction enzyme sites are underlined. for=forward primer; rev=reverse primer; RF=restriction-free; DR=direct repeats; IR=inverted repeats.

**Supplementary Table 2:** Oligonucleotides used for *in vitro* assays.

| **Oligonucleotide** | **Sequence** | **Figure** |
| --- | --- | --- |
| **Binding studies** | | |
| DR-top | AAGTGGTAAAAGTGATAAAAATG | 3c,d, 3-Sup1, 3-Sup2 |
| DR-bottom | CATTTTTATCACTTTTACCACTT | 3c,d, 3-Sup1, 3-Sup2 |
| ori-top | GATTAAGTGGTAAAAGTGATAAAAATGAAA | 5a, 5-Sup1 |
| ori-bottom | TTTCATTTTTATCACTTTTACCACTTAATC | 5a, 5-Sup1 |
| non-ori = T2 | GAGACCTACAGTAGTCGTAGCCACATCGCA | 5a,b,d, 5-Sup1 |
| T2-20 | CCACATCGCA | 5d |
| T2-15 | CGTAGCCACATCGCA | 5d |
| T2-10 | GTAGTCGTAGCCACATCGCA | 5d |
| T2-5 | CAACAGTAGTCGTAGCCACATCGCA | 5d |
| T2+5 | GAGACCAACAGTAGTCGTAGCCACATCGCAAGTGA | 5d |
| T2+10 | GAGACCAACAGTAGTCGTAGCCACATCGCAAGTGAACCTG | 5d |
| T2+15 | GAGACCAACAGTAGTCGTAGCCACATCGCAAGTGAACCTGACCTA | 5d |
| **Helicase assays** | | |
| HELA-5F (top) | GGATATGTATGGCATACAACTGGAAGTGGTAAGACGTTGACTTCTTTTAAA GCGAGTCAG | 3e |
| HELA-6R (bottom) | GACTGACGCTTTAAAAGAAGTCAACGTCTTACCACTTCCAGTTGTA TGCCATACATATCC | 3e |
| HELA-7R (bottom) | CTGACTCGCTTTAAAAGAAGTCAACGTCTTACCACTTCCAG TTGTATGCCATACATATCC | 3e |
| HELA-8R (bottom) | CGCTTTAAAAGAAGTCAACGTCTTACCACTTCCAGT TGTATGCCATACATATCC | 3e |
| HELA-9F (top) | GGATATGTATGGCATACAACTGGAAGTGGTAAGACG TTGACTTCTTTTAAAGCG | 3e |
| HELA-5F-AAGTG (top) | GGATATGTATGGCATACAACTGGTAGACGTAAGACGTTGACTTCTTTTAAAGCGAGTCAG | 3-Sup3 |
| HELA-6R-AAGTG (bottom) | GACTGACGCTTTAAAAGAAGTCAACGTCTTACGTCTACCAGTTGTATGCCATACATATCC | 3-Sup3 |
| **Primer extension assays** | | |
| Template | AATCTGACTCGCTTTAAAAGAAGTCAACGTCTTACCACGGCCAGTTGTATGCCAT | 4a |
| Primer* | ATGGCATACAACTGG | 4a |
| T1 | GTCGTAGCCACATCGCA | 4b |
| T2 | GAGACCTACAGTAGTCGTAGCCACATCGCA | 4b,c,d, 7b |
| P1 | GCGATGTGGCTA | 4b,d, 7b |
| T3 | CTCCAGACAAGGAAGTGGTAAAAGTGATAAAAATGGCTAGGCAGATCGCA | 4b |
| T4 | CTCCAGACAAGGCATTTTTATCACTTTTACCACTTGCTAGGCAGATCGCA | 4b |
| P2 | GCGATCTGCCTA | 4b |
| **Primase assays** | | |
| T2 | GAGACCTACAGTAGTCGTAGCCACATCGCA | 6a,b,c, 7c,d |
| P1 | GCGATGTGGCTA | 6a,b,c, 7d |
| M13_primer | CACACAGGAAACAGCTATGACCAT | 6c |
| Template 1 = T2 | GAGACCTACAGTAGTCGTAGCCACATCGCA | 8a |
| Template 2 | GCAGTTGCCTGTAGTACTCATATGATTAAG | 8a |
| Template 3 | CTTAATCATATGAGTACTACAGGCAACTGC | 8a |
| Template 4 | GATCGGATCCGGGTCACGTATCAAAGTGTC | 8a |
| Template 5 | GATCGGATCCATGAATCATATTTTAGAAATG | 8a |
| Template 6 | CAGTTTTGATATCAAATTATACATGTCAACG | 8a |
| Template 7 | GATCGGATCCCCCTGATTCTGTGGATAACCG | 8a |
| Template 8 | GATCGGATCCGATATCGAGTACCTCCAAAATG | 8a |
| poly-dT | TTTTTTTTTTTTTTTTTTTTTTTTTTTTTT | 8d |
| dT-ori8-dT | TTTTTTTTTTTACCACTTATTTTTTTTTTT | 8d |
| ori-top | TATGATTAAGTGGTAAAAGTGATAAAAATG | 8d |
| ori-bottom | CATTTTTATCACTTTTACCACTTAATCATA | 8d |
| **Reverse transcriptase assay** | | |
| RT-temp* | ATCAATAACCCCACTAACGCTTGCAGTATTGAAACTCA | 6d |
| RT-primer | TGAGTTTCAATACTGCAAG | 6d |

All sequences shown in 5’ to 3’ direction. *both DNA and RNA oligonucleotides were used.

**Supplementary Table 2:** Primase start sites identified in the study.

| **Site** | **Sequence** | **Comments** |
| --- | --- | --- |
| 1 | TCGTAGCCACATCG |  |
| 2 | CACATCGCAAGTGA |  |
| 3 | GTAGCCACATCGCA |  |
| 4 | AGTGAACCTGACCT |  |
| 5 | TCAACGTCTTACCA | Excluded from analysis in Fig 8b* |
| 6 | TTTACCACTTAATC |  |
| 7 | TGTAGTACTCATAT |  |
| 8 | AAGTCAACGTCTTA | Excluded from analysis in Fig 8b* |
| 9 | GTCAACGTCCCAGT | Excluded from analysis in Fig 8b* |
| 10 | GTCCCAGTTGTATT | Excluded from analysis in Fig 8b* |
| 11 | CAACGTCCCAGTTG | Excluded from analysis in Fig 8b* |
| 12 | GTCGTAGCCACATC |  |
| 13 | AGTACTACAGGCAA |  |
| 14 | GTACTACAGGCAAC |  |
| 15 | CACGTATCAAAGTG |  |
| 16 | CATATTTTAGAAAT |  |
| 17 | CATGAATCATATTT |  |
| 18 | AATTATACATGTCA |  |
| 19 | TATTCATCGTCAAA |  |
| 20 | TTCATCGTCAAAGT |  |
| 21 | TCATCGTCAAAGTC |  |
| 22 | AGTACCTCCAAAAT |  |
| 23 | GTACCTCCAAAATG |  |
| 24 | ACGGACATAGAAAA |  |
| 25 | GATACCCCCGAATT |  |
| 26 | ATACCCCCGAATTT |  |
| 27 | AACCTTCCCGGCTT |  |
| 28 | ACCTTCCCGGCTTC |  |
| 29 | ACCCCTCCAAATTC |  |
| 30 | CCCCTCCAAATTCA |  |
| 31 | GACAAGAGACAAAC |  |
| 32 | ACTTTGACGATGAA |  |
| 33 | TTTTACCCTTCATA |  |
| 34 | TACCCTTCATATCA |  |
| 35 | ACGACTACTGTAGG |  |
| 36 | AAAGTGATAAAAAG |  |
| 37 | CACTTTTACCACTT |  |
| 38 | ACTTTTACCACTTA |  |
| 39 | CTTTTACCACTTAT |  |

All sequences shown in 5’ to 3’ direction. *Sites were excluded from analysis of the distance to the 3’ end of the template since their position exceeded 30 nt from the end and second site closer to the 3’ end could be identified on the substrate.
